# PTSD symptom severity relates to cognitive and psycho-social dysfunctioning – a study with Congolese refugees in Uganda

**DOI:** 10.1080/20008198.2017.1283086

**Published:** 2017-02-14

**Authors:** Herbert E. Ainamani, Thomas Elbert, David K. Olema, Tobias Hecker

**Affiliations:** ^a^ Department of Psychology, Mbarara University of Science and Technology, Mbarara, Uganda; ^b^ Department of Psychology, Bishop Stuart University, Mbarara, Uganda; ^c^ Department of Psychology, University of Konstanz, Konstanz, Germany; ^d^ Department of Psychology, University of Zurich, Zurich, Switzerland

**Keywords:** Refugees, PTSD, cognitive functions, psycho-social functioning, DR Congo

## Abstract

**Background**: In the ongoing conflict in the Democratic Republic of the Congo (DRC), civilians have been heavily exposed to traumatic stressors. Traumatizing experiences cumulatively heighten the risk for trauma-related disorders, and with it affect cognitive and psycho-social functioning.

**Objectives**: We aimed at investigating the association between trauma-related disorders and cognitive and psycho-social functioning and hypothesized that PTSD symptom severity would negatively correlate with executive functioning, working memory and psycho-social functioning in everyday life.

**Method**: In total, 323 Congolese refugees (mean age: 31.3 years) who arrived in the Ugandan Nakivale refugee settlement after January 2012 were assessed regarding their exposure to traumatic events, PTSD symptom severity (posttraumatic symptom scale interview), executive functioning (Tower of London), working memory performance (Corsi block tapping task) and psycho-social dysfunctioning (Luo functioning scale).

**Results**: Hierarchical regression analyses indicated a significant negative association between PTSD symptom severity and working memory (β = –0.32, *p* < 0.001), as well as executive functions (β = –0.19, *p* = 0.003). Furthermore, the impairment of psycho-social functioning in everyday life was positively related with PTSD symptom severity (β = 0.70, *p* < 0.001), and negatively with executive functioning (β = –0.15, *p* = 0.003). However, working memory performance was not significantly related to psycho-social dysfunctioning (β = 0.09, *p* > 0.05).

**Conclusion**: Trauma survivors not only suffer from the core PTSD symptoms but also from impaired cognitive functioning. PTSD symptom severity seems furthermore to be related to impaired psycho-social functioning. Our findings suggest that trauma-related mental health problems may heighten the risk for poverty and lack of prospect and further aggravate the consequences of war and conflict.

## 1. Background

Forced migration is a major challenge for the international community. Recently, the United Nations High Commissioner for Refugees (UNHCR) announced a major increase in worldwide refugee numbers that has not been seen since the early 1990s (UNHCR, 2013). In the middle of 2013, 490,000 persons originating from the Democratic Republic of the Congo (DRC) were forced to seek refuge in neighbouring countries, many of them in Uganda (UNHCR, 2013). The DRC has been trapped in an ongoing cycle of war and violence for more than two decades. With the rise of the insurgency *Mouvement du 23 mars* in early 2012, the eastern DRC entered another period of high conflict intensity, which peaked when they took over the regional capital of Goma. Civilians suffer on a large scale from the consequences of violence (Elbert et al., 2013). Many of them have been affected by different types of traumatic events such as being exposed to severe physical and sexual violence, being threatened to be killed, witnessing loved ones being killed, being kidnapped, and being sexually abused. These traumatic experiences are emotionally shocking and detrimental to psychological wellbeing (Bastick, Grimm, & Kunz, 2007). As a consequence, many refugees suffer from stress- and trauma-related disorders, e.g. posttraumatic stress disorder (PTSD), substance abuse or depression, with prevalence rates for trauma-related disorders ranging from 20% to more than 50% depending on the conflict setting (Catani, Jacob, Schauer, Kohila, & Neuner, 2008; Hecker, Fetz, Ainamani, & Elbert, 2015; Onyut et al., 2009). Studies in war and crisis regions have repeatedly shown a strong association between the number of traumatic event types that an individual has been exposed to and the development of PTSD (Neuner et al., 2004a; Kolassa et al., 2010; Wilker et al., 2015). Thus, the risk for trauma-related disorders increases with the number of experienced traumatic event types and may explain the high and also varying PTSD prevalence rates in conflict and crisis regions. This cumulative effect of trauma exposure has often been described as the *building block effect* (Neuner et al., 2004a; Steel, Silove, Phan, & Bauman, 2002; Wilker et al., 2015). Yet, the consequences of trauma exposure and high PTSD prevalence in a population of refugees may go beyond individual suffering and may impact the livelihoods of family members and entire communities due to impaired psycho-social functioning in everyday life (Neuner, Schauer, Klaschik, Karunakara, & Elbert, 2004b).

Beside trauma-related psychopathological symptoms, impaired cognitive functions may explain impaired psycho-social functioning in everyday life. A few studies on cognitive impairment due to trauma-related disorders mostly from western countries have not yielded consensus about which cognitive domains are mostly affected by trauma-related disorders. However, impairments have been identified in almost all domains of cognitive functioning, including working memory, executive functions, intellectual functioning, concentration, learning and attention (Bremner et al., 2004; De-Prince, Wenzierl, & Combs, 2009; Leskin & White, 2007; Samuelson et al., 2006; Stein, Kennedy, & Twamley, 2002; Vasterling et al., 2002). For example, Twamley et al. (2009) found poor performance in executive functions among women with PTSD compared to women without PTSD. Similarly, also in various other studies PTSD has been associated with poor performance in executive functioning (e.g. Kanagaratnam & Asbjornsen, 2007; Stein et al., 2002). Furthermore, in a study that compared children exposed to traumatic events and those that were not, a link between trauma exposure and poor performance of executive functioning was observed (De Prince et al., 2009). Although the available literature suggests that individuals with PTSD often exhibit deficits in executive functions, deficits in problem solving, inhibition, interference control and non-verbal working memory are also related to exposure to traumatic events and PTSD (Beers & De Bellis, 2002; Bremner et al., 2003; Morgan, Doran, Steffian, Hazlett, & Southwick, 2006). For example, findings indicated that exposure to traumatic stressors was related to inaccuracy of working memory (Schweizer & Dalgleish, 2011). Similarly, McNally (2006) observed poor performance in concentration, learning, and working memory among individuals with PTSD.

Only very few studies thus far investigated cognitive functioning in (post-) conflict areas or among traumatized refugees. For example, in a study with traumatized refugees, participants with PTSD scored significantly lower in executive functions, attention, visual spatial functions, and working memory (Kivling-Boden & Sundbom, 2003). Though previously findings consistently suggested that PTSD symptom severity is likely to be a strong predictor of deficits in cognitive functions, it is not known whether these cognitive impairments have negative implications for psycho-social functioning in everyday life beyond the impact of poor mental health.

### 1.1. Objectives

Refugees that still live in a region of ongoing war and conflict – such as Congolese refugees in Uganda – are at high risk to experience traumatic events and to develop trauma-related disorders such as PTSD. Besides suffering from the core PTSD symptoms, previous research from non-conflict settings suggests that traumatized refugees may also suffer from impaired cognitive functioning as a probable consequence of PTSD symptom severity. The scope of the present study is, however, broader, as we investigate whether trauma-related disorders and impaired cognitive functioning may contribute to poverty and lack of prospect through an impairment of psycho-social functioning in everyday life, such as for work-related tasks, household chores, or maintaining social relationships. We hypothesized that PTSD symptom severity would be negatively correlated with both (a) working memory performance and (b) executive functioning. Furthermore, we expected (c) an association between impaired psycho-social functioning in everyday life and PTSD symptom severity, impaired executive functioning, and poor working memory performance.

## 2. Methods

### 2.1. Participants

Between March and June 2013 a sample of 323 persons were interviewed in Nakivale Refugee Settlement in Western Uganda. At the time of investigation, this settlement had received a high influx of refugees from the DRC. In March 2014 the population of the settlement was 60,992 persons, of which 32,455 were Congolese (UNHCR Uganda, 2014). Only refugees from the DRC who arrived in the settlement after January 2012 were interviewed. This inclusion criterion was validated at the beginning of each interview. Of the included sample, 182 (56%) were female. Mean age was 31.28 years (SD = 9.03) with an age range of 18 to 65. All of them reported having fled from Eastern DRC because of the threats they were facing due to the conflict in their home country.

### 2.2. Procedure

Three psychologists and a social scientist conducted the semi-structured interviews. All interviewers were extensively trained in psychological assessment. The interviewers practiced the assessment in joint interviews to accomplish high inter-rater reliability. All interviews were conducted in Swahili, which is the lingua franca both in the Eastern DRC and in Nakivale refugee camp. One interviewer was fluent in Swahili. Hence, 120 interviews were conducted only in Swahili. The others were translated from English to Swahili by two well-trained interpreters.

The interviews took place in different parts of the refugee settlement. We first contacted the local authorities of the settlement’s subdivisions. After giving their consent, the authorities announced our arrival in their communities and gathered persons that fulfilled our inclusion criteria, i.e. being Congolese, being 18 years or older, having fled from DRC after January 2012. Using snowball sampling we returned to the respective places until no more persons fulfilling these inclusion criteria appeared. Practically, we worked together with the subdivisions’ chairpersons and asked them to inform all Congolese refugees who had arrived recently about the study. Furthermore, we asked all participants to spread the information to other refugees that had recently arrived from the DRC. We then went to the next subdivision where recent arrivals were living. We collected oral and written informed consent from the participants to ensure comprehension and full awareness of the content. Illiterate participants gave their fingerprints instead of signature. Each interview was conducted in a calm and discrete setting. At the end of the interview, each participant received a bar of soap and a package of salt as compensation. The Institutional Review Board of the Mbarara University of Science and Technology as well as the Ugandan National Council for Science and Technology approved this study.

### 2.3. Measures

All assessment instruments were applied as semi-structured interviews. After informed consent, we assessed socio-demographic information (e.g. age, sex and educational background).

#### 2.3.1. Traumatic and other adverse experiences

Exposure to traumatic and other adverse experiences was assessed using a checklist of 30 war- and non-war-event types (e.g. natural catastrophes, physical assault, sexual assault) and 21 items related to exposure to family and community violence. This checklist was an adapted version of a checklist by Neuner et al. (2004a), which had previously shown high test–retest reliability and significant accordance with the event list of the composite international diagnostic interview in a study in Uganda (Ertl et al., 2010). It has also been successfully used in studies in the Great Lakes Region (e.g. Hermenau, Hecker, Schaal, Maedl, & Elbert, 2013) and with Congolese refugees in Nakivale refugee camp (Hecker et al., 2015). The number of times a specific event had been experienced was not assessed, as measuring event types provide an accurate and practical measure of traumatic experiences (Wilker et al., 2015). For the analysis, we calculated a sum score of lifetime exposure to traumatic and adverse experiences by summing up all items (range: 0–51).

#### 2.3.2. PTSD symptom severity

The PTSD symptom scale – interview (PSS-I; Foa, Riggs, Dancu, & Rothbaum, 1993) was utilized to determine PTSD symptom severity. The 17 DSM-IV symptom criteria for PTSD are assessed with one question for each symptom and refer to the previous two weeks. The answers were coded on a four-point scale ranging from *not at all* (0) to *five or more times per week/very much* (3). The PSS-I has been shown to have good psychometric properties (e.g. Cronbach’s α = 0.86, inter-rater reliability = 0.93; Foa & Kozak, 1986). The instrument has been validated for use in Uganda (Ertl et al., 2010) and has been successfully used in the DRC (e.g. Hecker, Hermenau, Maedl, Schauer, & Elbert, 2013) and with Congolese refugees in Nakivale refugee camp (Hecker et al., 2015). We computed dimensional PTSD severity scores by adding the scores of each question, resulting in a sum score ranging from 0 to 51. Cronbach’s alpha coefficient in the present sample was 0.96.

#### 2.3.3. Impairment of psycho-social functioning in everyday life

To assess functional impairment in specific daily routines, six items concerning possible deficits in psycho-social areas (e.g. home management, work or leisure activities) were answered on a five-point Likert scale ranging from 0 (*none*) to 4 (*often cannot do it*). The items were based on the Luo functioning scale (LFS, Ertl et al., 2010). The LFS has been used in numerous studies in the Great Lakes Region and has proven high construct validity (Ertl et al., 2010). For the analysis, we computed a sum score of all six items (possible range: 0–24). In the present sample, Cronbach’s α coefficient was 0.86.

#### 2.3.4. Working memory

We assessed working memory using the Corsi block tapping (CBT) task. This neuropsychological test has been widely used as a measure of spatial memory in both clinical and experimental contexts for several decades, is the most important nonverbal task in neuropsychological research, and comes with good psychometric properties shown in validation studies (Berch, Krikorian, & Huha, 1998; Kessels, Van Zandvoort, Postma, Kappelle, & Haan, 2000). It has also been successfully used in studies in the Great Lakes Region (e.g. Hecker, Hermenau, Salmen, Teicher, & Elbert, 2016). The task requires participants to reproduce block-tapping sequences of increasing length in the same or in the reversed order and provides an index of working memory capacity. The Corsi apparatus consisted of nine 2.25 cm^3^ black, wooden blocks fixed to a 27.5 cm × 22.8 cm grey, wooden board. The blocks were placed as described in the original test developed by Corsi (Berch et al., 1998; Kessels et al., 2000). Each cube was numbered on one side so that the numbers were visible to the interviewer but not to the participant. The participant was seated in front of the interviewer, who subsequently tapped the blocks starting with a sequence of three blocks. Three trials were given per block sequence of the same length. The blocks were touched with the index finger at a rate of approximately one block per second with no pauses between the individual blocks. In the first application of the test after the first half of the interview, the participant had to tap the block sequences in the same order immediately after the interviewer was finished. In the second application at the end of the interview, the participants had to tap the block sequence in reversed order. We computed a total score for both applications (same order and reversed order) by adding the number of correctly repeated sequences until the test was discontinued (i.e. the number of correct trials). The total score ranged from 0 to 21. High performance implied higher working memory capacity.

#### 2.3.5. Executive functions

The Tower of London (TOL) was used to assess executive functions, such as planning and problem solving (Krikorian, Bartok, & Gay, 1994). The TOL is a classic neuropsychological test for the assessment of executive functions that include planning and problem-solving skills, which has been widely used in diverse cultures (Agranovich, Panter, Puente, & Touradji, 2011; Lam et al., 2013). The validation study revealed sound psychometric properties (Orsini, 1994). The TOL consisted of three wooden pegs, which were fixed on a block of wood and three wooden balls of different colours (black, grey and white) that were placed on the pegs and moved from one peg to another. The participants were shown 12 pictures which depicted the TOL with the balls being placed in different positions on the pegs and were asked to arrange the balls to match the positions on the picture. Each trial started from the same starting positions and varied in difficulty due to the number of moves that were allowed to arrange the balls to match the picture (from two to five). Three attempts were granted for each problem. For each problem up to three points could be earned (if successful in the first attempt). The total sum scores ranged from 0 to 36. Higher grades would mean better performance.

### 2.4. Data analysis

Of the 323 interviews, 16 could not be completed due to logistical reasons. These incomplete interviews were excluded from all analyses due to missing data. The association between the number of traumatic event types, PTSD symptom severity, and working memory was analysed using hierarchical multiple linear regression. The regression models fulfilled all the necessary quality criteria for linear regression analysis. The residuals did not deviate significantly from normality (Kolmogorow–Smirnov-*Z* = 0.58, *p* = 0.887), linearity or homoscedasticity. No univariate outliers could be identified. However, Cook’s distance revealed nine multivariate outliers (2 SD > M; Hecker et al., 2013). Consequently, these multivariate outliers were excluded from this analysis. The maximum variance inflation factor did not exceed 1.68. Hence, we did not need to take multicollinearity into account.

Also, the association between the number of traumatic event types, PTSD symptom severity, and executive functions was analysed using hierarchical multiple linear regression. Again the regression models fulfilled all the necessary criteria for linear regression analysis. The residuals did not deviate significantly from normality (Kolmogorow–Smirnov-*Z* = 1.62, *p *> 0.01), linearity or homoscedasticity. No univariate could be identified. However, Cook’s distance revealed 11 multivariate outliers. These multivariate outliers were excluded from this analysis. The maximum variance inflation factor did not exceed 1.64. Hence, we did not need to take multicollinearity into account.

The association between PTSD symptom severity, cognitive functions, and psycho-social dysfunctioning was analysed using a moderated sequential multiple regression. To mitigate multicollinearity, the predictor variables were mean-centred for calculations of interaction terms (Kleinbaum, Kupper, Nizam, & Muller, 2008). The regression models fulfilled all the necessary criteria for linear regression analysis. The residuals did not deviate significantly from normality (Kolmogorow–Smirnov-*Z* = 0.56, *p =* 0.792), linearity or homoscedasticity. No univariate could be identified. However, Cook’s distance revealed 14 multivariate outliers. These outliers were excluded from this analysis. The maximum variance inflation factor did not exceed 1.52. Hence, we did not need to take multicollinearity into account. Our metric for a small effect size was *f* ^2^ ≥ 0.02, for a medium effect *f* ^2^ ≥ 0.15, and for a large effect *f* ^2^ ≥ 0.35. All analyses used a two-tailed α = 0.05. Statistical analyses were conducted using SPSS for Mac Version 22 (SPSS Inc, IBM, IL, USA).

## 3. Results

Table 1 displays the descriptive statistics of the main variables. Participants were exposed to high levels of traumatic experiences. The results revealed that female respondents presented higher PTSD symptom severity but not a higher exposure to traumatic experiences than their male counterparts. Female participants also showed higher impairment in psycho-social functioning in everyday life. Compared to males, female participants performed poorer on both the CBT task and the TOL test.

**Table 1.** Descriptive statistics of the main study variables and gender differences.

		Males		Females		
	N	*M*	*SD*	*M*	*SD*	*t*
Age	M = 141; F = 182	31.94	9.03	30.87	9.01	1.06
Education	M = 139; F = 180	7.06	4.73	5.38	4.81	3.12**
Traumatic exposure ^a^	M = 141; F = 179	34.64	8.02	36.25	8.03	−1.78
PTSD symptom severity ^b^	M = 141; F = 179	29.71	13.90	35.70	11.64	−4.11***
Psycho-social dysfunctioning in everyday life ^c^	M = 141; F = 181	5.86	5.32	8.17	6.05	−3.64***
Corsi block tapping (CBT) task	M = 141; F = 179	12.21	4.10	10.23	3.71	4.53***
Tower of London (TOL) test	M = 140; F = 179	27.71	6.13	24.74	6.85	4.02***

t: t-test or Welch-test statistics; *p ≤ 0.05. **p ≤ 0.01. ***p ≤ 0.001. ^a^Index of traumatic and other adverse event types, ^b^PSS-I sum score, ^c^Luo functioning scale sum score.

### 3.1. Association between PTSD symptom severity and working memory performance

The first regression model with gender, age and education as control variables was significant and explained 15% of the variability of the working memory score (adj. *R* ^2^ = 0.15, *F*(3, 296) = 18.06, *p* < 0.001, *f* ^2^ = 0.18). Adding the number of traumatic and adverse events types and PTSD symptom severity as additional predictors improved the model significantly (Δ*R* = 0.07, *F*(2, 294) = 12.56, *p *< 0.001, *f* ^2^ = 0.08). The second model explained 21% of the variability of working memory. Our analysis revealed a negative association between PTSD symptom severity and working memory capacity. However, exposure to traumatic and adverse experiences was not significantly related to the working memory performance. Female gender was negatively related to working memory capacity and years of formal education correlated positively with working memory performance (Table 2).

**Table 2.** Results of regression analysis predicting working memory.

	Scores of CBT			
	*B*	*SE*	β	*T*
Step 1				
Sex	−1.79	0.42	−0.23	−4.26***
Age	−0.01	0.02	−0.02	−0.30
Education	0.22	0.04	0.28	5.05***
Step 2				
Sex	−1.21	0.42	−0.16	−2.87**
Age	−0.02	0.02	−0.05	−0.88
Education	0.25	0.04	0.31	5.81***
PTSD symptom severity ^a^	−0.10	0.02	−0.32	−4.85***
Trauma exposure ^b^	0.06	0.03	0.11	1.71

R^2^adj = 0.21, *f* ^2^ = 0.27, *n* = 300. *B*: unstandardized regression weight; *SE*: standard error; *β*: standardized regression weight; *T* = t test statistics; **p ≤ 0.01. ***p ≤ 0.001. ^a^ PTSD symptom scale – interview sum score, ^b^ Index of traumatic and other adverse event types.

### 3.2. Association between PTSD symptoms severity and executive functioning

The first regression model including gender, age and education as control variables was significant and explained 24% of the variability of executive functions (adj. *R* ^2^ = 0.24 *F*(3, 294) = 32.84, *p* < 0.001, *f* ^2^ = 0.32). Adding the number of traumatic and adverse events types and PTSD symptom severity as additional predictors improved the model significantly (ΔR = 0.02, *F*(2, 292) = 4.40, *p *< 0.05, *f* ^2^ = 0.02). The second model explained 26% of the variability of executive functions. Our analysis revealed a negative association between scores of PTSD symptom severity and executive functioning. Again, exposure to traumatic and adverse experiences was not significantly related to executive functioning. Female gender and age was negatively related to executive functioning. Years of formal education were positively related to executive functioning (Table 3).

**Table 3.** Results of regression analysis predicting executive functions.

	TOL score			
	*B*	*SE*	β	*T*
Step 1				
Sex	−2.37	0.61	−0.20	−3.85***
Age	−0.07	0.04	−0.11	−2.07*
Education	0.51	0.06	0.41	8.04***
Step 2				
Sex	−1.91	0.63	−0.16	−3.02**
Age	−0.08	0.04	−0.12	−2.35*
Education	0.52	0.06	0.43	8.29***
PTSD symptom severity ^a^	−0.09	0.03	−0.19	−2.95**
Trauma exposure ^b^	0.07	0.05	0.09	1.41

R^2^adj = 0.26, *f* ^2^ = 0.35, *n* = 298. *B*: unstandardized regression weight; *SE*: standard error; *β*: standardized regression weight; *T* = t test statistics; *p ≤ 0.05. **p ≤ 0.01. ***p ≤ 0.001. ^a^PTSD symptom scale – interview sum score, ^b^Index of traumatic and other adverse event types.

### 3.3. PTSD symptoms severity and cognitive functioning relates to psycho-social dysfunctioning

The first regression model including gender, age and education as control variables was significant but explained only 7% of the variance of psycho-social dysfunctioning (adj. *R* ^2^ = 0.07 *F*(3, 291) = 8.31, *p* < 0.001, *f* ^2^ = 0.08). Adding PTSD symptom severity as additional predictor improved the model fit significantly (ΔR = 0.41, *F*(1, 290) = 236.14, *p *< 0.001, *f* ^2^ = 0.70). With 48% the second model explained a substantial portion of the variance of psycho-social dysfunctioning. Adding working memory performance and executive functioning as additional predictors improved the model even further (ΔR = 0.02, *F*(2, 288) = 5.71, *p *= 0.004, *f* ^2^ = 0.02). This model explained 50% of the variability of psycho-social dysfunctioning. Adding the interaction terms of working memory performance and PTSD symptoms severity as well as of executive functioning and PTSD symptoms severity improved the model further (ΔR = 0.01, *F*(2, 286) = 3.88, *p* = 0.022, *f* ^2^ = 0.01). The final model explained 51% of the variability of psycho-social dysfunctioning. Our analysis revealed a strong positive association between the scores of PTSD symptom severity and psycho-social dysfunctioning as well as a small negative relation between executive functioning. PTSD symptom severity moderated this relationship; the negative relation between executive functioning and psycho-social dysfunctioning was stronger for those who reported high PTSD symptom severity. In contrast, working memory performance was not significantly related to psycho-social dysfunctioning, neither was this relationship moderated by PTSD symptom severity. Furthermore, older age and years of formal education were positively related to psycho-social dysfunctioning (Table 4).

**Table 4.** Results of regression analysis predicting psycho-social functioning in everyday life.

	LFS score ^a^			
	*B*	*SE*	β	*T*
Step 1				
Sex	2.86	0.67	0.25	4.27***
Age	0.04	0.04	0.06	1.02
Education	0.24	0.07	0.20	3.41**
Step 2				
Sex	0.77	0.52	0.07	1.49
Age	0.12	0.03	0.17	4.02***
Education	0.11	0.05	0.09	2.07*
PTSD symptom severity ^a^	0.31	0.02	0.68	15.37***
Step 3				
Sex	0.69	0.52	0.06	1.34
Age	0.10	0.03	0.16	3.66***
Education	0.15	0.06	0.12	2.53*
PTSD symptom severity ^a^	0.32	0.02	0.69	15.32***
Executive functioning	−0.14	0.04	−0.15	−3.13**
Working memory performance	0.15	0.07	0.10	2.09*
Step 4				
Sex	0.49	0.52	0.04	0.94
Age	0.10	0.03	0.14	3.39***
Education	0.15	0.06	0.12	2.57*
PTSD symptom severity ^a^	0.32	0.02	0.70	15.41***
Executive functioning	−0.13	0.04	−0.15	−3.02**
Working memory performance	0.13	0.07	0.09	1.81
ExF*PSS	−0.01	0.01	−0.13	−2.70**
WMP*PSS	0.01	0.01	0.10	1.93

R^2^adj = 0.51, *f* ^2^ = 1.04, *n* = 295. *B*: unstandardized regression weight; *SE*: standard error; *β*: standardized regression weight; *T* = t test statistics; ExF*PSS: interaction between executive functioning and PTSD symptom severity; WMP*PSS: interaction between working memory performance and PTSD symptom severity. *p ≤ 0.05. **p ≤ 0.01. ***p ≤ 0.001. ^a^Luo functioning scale, ^b^PTSD symptom scale – interview sum score.

## 4. Discussion

The aim of the present study was to examine the association between PTSD symptom severity, executive functioning, working memory performance, and psycho-social dysfunctioning in everyday life in a sample of Congolese refugees in Uganda. Our sample was highly exposed to traumatic experiences. Though female participants did not experience more traumatic events, they reported higher PTSD symptom severity. This may be explained by the exposure to specific types of traumatic events, for example, 56% of the woman (compared to 15% of the man) reported having been raped. It is well known that the conditional prevalence of PTSD after exposure to sexual violence is particularly high (Schalinski, Elbert, & Schauer, 2011).

Our results revealed a negative relationship between PTSD symptom severity and working memory performance after controlling for the potential influences of age, gender and educational background. Though this effect was small, our findings are consistent with our hypothesis and a number of reports suggesting that individuals with PTSD have a smaller working memory capacity (Elbert et al., 2009; Johnsen, Kanagaratnam, & Asbjrnsen, 2008; Kivling-Boden & Sundbom, 2003; Samuelson et al., 2006; Yehuda, Golier, & Halligan et al., 2004). In addition, we found a negative association between PTSD symptom severity and executive functioning after controlling for the potential influences of age, gender and educational background. Though this effect was also small, this observation is in accord with our hypothesis and prior findings showing a negative relationship between PTSD and executive functions following the exposure to multiple traumatic events (Bremner & Narayan, 1998; Bremner & Vermetten, 2001; DeBells, 2005; Teicher et al., 2003; Twamley et al., 2009). The observed negative relationship between PTSD symptom severity and cognitive functioning provides further support to the notion that trauma survivors suffer besides trauma-related mental health problems also from impaired cognitive functioning. This is the first study – that we are aware of – that reports these relations in a conflict setting. The previous studies reported this relation mostly in Western samples and after non-war related trauma exposure, in particular after exposure to childhood trauma (Kanagaratnam & Asbjornsen, 2007; Stein et al., 2002). However, we did not systematically assess adverse childhood experiences. Yet, it is known that these increase the vulnerability for PTSD (Nandi, Crombach, Bambonye, Elbert, & Weierstall, 2015) and thus could drive both trauma-related psychopathology and neuropsychological shortfalls. Further studies would need to assess the adverse childhood experiences, ideally with a record of the timing, as type and timing may both be relevant in increasing the vulnerability for psychopathological development and for specific neurocognitive deficits.

We chose measures that do not require literacy. Yet, cognitive variables, such as working memory and executive functioning, are, as is to be expected, highly correlated with educational level. Nevertheless, PTSD symptom severity explained an additional, albeit smaller proportion of the variability of working memory performance (7%) and executive functioning (2%). Most likely both the lower educational level and the higher PTSD symptom severity of female participants may best explain the gender differences (e.g. Onyut et al., 2009).

Furthermore, in the present study we investigated the association between PTSD symptom severity, cognitive functioning, and the impairment of psycho-social functioning in everyday life. Besides trauma-related symptoms, impaired cognitive functions may also be one explanation for impaired psycho-social functioning in everyday life. In accordance with our hypothesis and prior studies (Martinez-Aran et al., 2007; Mora, Portella, Forcada, Vieta, & Mur, 2013; Ogle, Rubin, & Siegler, 2013), we found that PTSD symptoms severity was strongly related to impairments of psycho-social dysfunctioning after controlling for the influences of age, gender and education. Above the influence of PTSD symptom severity, executive functioning was negatively related to psycho-social dysfunction. This relation was also moderated by PTSD symptom severity, revealing that for participants with high levels of PTSD symptoms this relation was stronger. On the other hand, working memory performance was not related to impaired psycho-social functioning in everyday life. Our analyses revealed a large effect for the association between PTSD symptom severity and psycho-social dysfunctioning in everyday life. Yet, the effect size of the negative association between psycho-social dysfunctioning in everyday life and executive functioning was small and in general the findings for cognitive functions inconsistent. Our findings are only partly consistent with studies that have reported strong associations between trauma-related disorders, cognitive dysfunctions and a wide-range of physical and psycho-social dysfunctions, e.g. work-related problems (Johnsen, Laberg, Matthiessen, Dyregrov, & Dyregrov, 2015; Ogle et al., 2013; Sanderson & Andrews, 2006; Sandström et al., 2011; Wilson et al., 2006). Especially in settings of war and conflict, impairments in psycho-social functioning may aggravate poverty and lack of prospect. Thus, our findings support the notion that the consequences of trauma exposure and high PTSD prevalence in a population of refugees may go beyond individual suffering and may impact the livelihoods of family members and whole communities due to impaired psycho-social functioning in everyday life (Neuner et al., 2004b).

### 4.1. Clinical implications and future research

Frequent and severe exposure to violence may not only heighten the risk of trauma-related and other mental health problems, but also seems to affect people’s cognitive and psycho-social functioning. The combination of trauma-related symptoms (PTSD, depression, alcohol abuse, enhanced aggressive behaviour) and psycho-social functioning may contribute to the elevated levels of poverty and lack of prospects in post-conflict societies. Yet, this hypothesis needs to be tested in future studies. Nonetheless, our findings underline the need for mental health policies and services to also implement trauma-focused treatment for refugees living in unsafe conditions. Neuner et al. (2004b), for example, showed that narrative exposure therapy (NET) is successful in treating trauma-related disorders in refugees living under such circumstances. In their study, 62% of the treatment group had left the refugee settlement one year after treatment, whereas only 7% (supportive counselling) and 17% (psycho-education) respectively of the control groups. Refugees who left the settlement often moved to safer and more fertile places in self-settlements, or sought jobs in the surrounding region. Neuner et al. (2004b) concluded that trauma-focused treatment, i.e. NET, did not only improve trauma-related symptoms but also psycho-social functioning in everyday life. In line with this, Hall et al. (2014) found that cognitive processing therapy increased social capital among survivors of sexual violence in the low-income conflict-affected context of the Eastern DRC. These and the results of this study suggest that trauma survivors would not only benefit from trauma-focused treatment in terms of improved mental health but also in terms of less trauma-related impairments in psycho-social functioning. This underlines the importance of providing traumatized refugees with trauma-focused treatment options to support both individual suffering and the livelihoods of the entire family and whole communities.

### 4.2. Limitations

There are some limitations that should be noted. The cross-sectional study design did not allow for the establishment of directionality. For example, it remains unclear whether PTSD symptom severity causes deficits in cognitive domains of working memory and executive functions, whether individuals with cognitive deficits are more likely to develop trauma-related disorders, or both are driven by other factors like adverse childhood experiences. Longitudinal and prospective studies are needed to shed light on the causal relations. Furthermore, with our snowball sampling approach we cannot completely rule out a selection bias. Also, other factors, such as malnutrition or sleep deprivation, may have influenced the cognitive functioning at the time of assessment. This explains also why the regression analysis explained only 21% and 26% of the variability of working memory performance and executive functioning, respectively. This study does not aim to explain the entire variability of the cognitive functioning, but the particular influence of PTSD symptom severity. The latest version of the DSM had been published shortly before we conducted the study. Yet, at the time of assessment no instrument that had been validated in a comparable sample was available. We therefore used the well-validated PSS-I that was still based on DSM-IV. Furthermore, this is the first study that applied the TOL test for executive functions in a sample in the Great Lakes Region. Though the application was feasible, findings should be interpreted as explanatory. Generally, the participants talked very openly about their experiences and feelings. However, potential biases, such as social desirability, can never be ruled out for subjective reports.

## 5. Conclusions

The negative association between PTSD symptom severity and cognitive functioning indicated that trauma survivors – besides their PTSD core symptomatology – might also suffer from impaired cognitive functioning. Trauma-related mental health problems were strongly associated with impaired psycho-social functioning in everyday life. Both may contribute to poverty and lack of prospect in settings of war and conflict.
